# Supplementary material for: Meta-Analysis and Gene Set Enrichment Relative to ER Status Reveal Elevated Activity of MYC and E2F in the “Basal” Breast Cancer Subgroup
Source: PLoS One. 2009 Mar 9;4(3):e4710. doi: 10.1371/journal.pone.0004710 (PMC2650420; doi:10.1371/journal.pone.0004710)
Supplement: Dataset S1 — GSEA results from first screen. The file contains a zipped archive of the HTML results of the second GSEA screen. Reports for enriched or depleted datasets may be accessed from the “index.html” link within the my_analysis.GseaPreranked.1219975950625.rpt.zip ZIP archive. Enrichment plots are available for each of the 50 most enriched and depleted gene sets. (13.32 MB ZIP) [file pone.0004710.s001.zip › my_analysis.GseaPreranked.1219975950625.rpt.zip!//CHANG_SERUM_RESPONSE_UP.html]

Details for gene set CHANG\_SERUM\_RESPONSE\_UP[GSEA]

|  || Dataset | zSco.ERneg.to.ERpos\_collapsed |
| Phenotype | NoPhenotypeAvailable |
| Upregulated in class | na\_pos |
| GeneSet | CHANG\_SERUM\_RESPONSE\_UP |
| Enrichment Score (ES) | 0.48739758 |
| Normalized Enrichment Score (NES) | 2.1309662 |
| Nominal p-value | 0.0 |
| FDR q-value | 7.2363815E-5 |
| FWER p-Value | 0.0030 |
Table: GSEA Results Summary

  

Fig 1: Enrichment plot: CHANG\_SERUM\_RESPONSE\_UP      
 Profile of the Running ES Score & Positions of GeneSet Members on the Rank Ordered List

  

| PROBE | GENE SYMBOL | GENE\_TITLE | RANK IN GENE LIST | RANK METRIC SCORE | RUNNING ES | CORE ENRICHMENT || 1 | MSN |  |  | 20 | 6.766 | 0.0195 | Yes |
| 2 | EIF4EBP1 |  |  | 65 | 6.108 | 0.0351 | Yes |
| 3 | GGH |  |  | 161 | 5.504 | 0.0450 | Yes |
| 4 | RFC3 |  |  | 164 | 5.493 | 0.0619 | Yes |
| 5 | COTL1 |  |  | 178 | 5.444 | 0.0778 | Yes |
| 6 | CHEK1 |  |  | 253 | 5.088 | 0.0880 | Yes |
| 7 | MCM7 |  |  | 289 | 4.970 | 0.1007 | Yes |
| 8 | CORO1C |  |  | 299 | 4.934 | 0.1154 | Yes |
| 9 | LMNB2 |  |  | 345 | 4.820 | 0.1269 | Yes |
| 10 | SNRPA |  |  | 368 | 4.749 | 0.1400 | Yes |
| 11 | MYBL2 |  |  | 418 | 4.629 | 0.1506 | Yes |
| 12 | SRM |  |  | 435 | 4.575 | 0.1636 | Yes |
| 13 | SNRPD1 |  |  | 440 | 4.552 | 0.1775 | Yes |
| 14 | ENO1 |  |  | 465 | 4.475 | 0.1895 | Yes |
| 15 | TOMM40 |  |  | 561 | 4.216 | 0.1954 | Yes |
| 16 | TUBA1 |  |  | 563 | 4.214 | 0.2084 | Yes |
| 17 | PLAUR |  |  | 604 | 4.138 | 0.2182 | Yes |
| 18 | SLC16A1 |  |  | 638 | 4.055 | 0.2283 | Yes |
| 19 | MCM3 |  |  | 656 | 4.019 | 0.2395 | Yes |
| 20 | ADAMTS1 |  |  | 659 | 4.017 | 0.2518 | Yes |
| 21 | DCLRE1B |  |  | 696 | 3.939 | 0.2613 | Yes |
| 22 | SNRPB |  |  | 805 | 3.771 | 0.2648 | Yes |
| 23 | C1ORF33 |  |  | 831 | 3.737 | 0.2745 | Yes |
| 24 | PTS |  |  | 884 | 3.648 | 0.2819 | Yes |
| 25 | PPIH |  |  | 943 | 3.562 | 0.2886 | Yes |
| 26 | RNF138 |  |  | 1042 | 3.421 | 0.2917 | Yes |
| 27 | NUTF2 |  |  | 1044 | 3.419 | 0.3023 | Yes |
| 28 | SNRPA1 |  |  | 1064 | 3.399 | 0.3114 | Yes |
| 29 | PSMD14 |  |  | 1069 | 3.392 | 0.3216 | Yes |
| 30 | BRCA2 |  |  | 1126 | 3.331 | 0.3277 | Yes |
| 31 | SMURF2 |  |  | 1129 | 3.330 | 0.3379 | Yes |
| 32 | PFKP |  |  | 1165 | 3.281 | 0.3454 | Yes |
| 33 | CENPJ |  |  | 1172 | 3.270 | 0.3551 | Yes |
| 34 | EIF4G1 |  |  | 1224 | 3.214 | 0.3612 | Yes |
| 35 | TPI1 |  |  | 1247 | 3.192 | 0.3695 | Yes |
| 36 | ITGA6 |  |  | 1288 | 3.149 | 0.3762 | Yes |
| 37 | KIAA0090 |  |  | 1297 | 3.140 | 0.3853 | Yes |
| 38 | UBE2J1 |  |  | 1309 | 3.129 | 0.3942 | Yes |
| 39 | SFRS10 |  |  | 1320 | 3.118 | 0.4031 | Yes |
| 40 | CDCA4 |  |  | 1427 | 3.018 | 0.4045 | Yes |
| 41 | TPM2 |  |  | 1506 | 2.938 | 0.4077 | Yes |
| 42 | RUVBL1 |  |  | 1508 | 2.934 | 0.4167 | Yes |
| 43 | SLC25A5 |  |  | 1599 | 2.850 | 0.4187 | Yes |
| 44 | LOC56902 |  |  | 1605 | 2.845 | 0.4272 | Yes |
| 45 | EPHB1 |  |  | 1675 | 2.780 | 0.4306 | Yes |
| 46 | MYBL1 |  |  | 1787 | 2.704 | 0.4305 | Yes |
| 47 | FLJ10292 |  |  | 1898 | 2.608 | 0.4303 | Yes |
| 48 | SNRPC |  |  | 1939 | 2.581 | 0.4353 | Yes |
| 49 | NUDT1 |  |  | 1996 | 2.533 | 0.4389 | Yes |
| 50 | VDAC1 |  |  | 2049 | 2.484 | 0.4426 | Yes |
| 51 | RPN1 |  |  | 2053 | 2.479 | 0.4501 | Yes |
| 52 | H2AFZ |  |  | 2105 | 2.447 | 0.4538 | Yes |
| 53 | MET |  |  | 2188 | 2.394 | 0.4550 | Yes |
| 54 | MT1F |  |  | 2350 | 2.285 | 0.4499 | Yes |
| 55 | STK17A |  |  | 2359 | 2.282 | 0.4564 | Yes |
| 56 | CCT5 |  |  | 2398 | 2.262 | 0.4605 | Yes |
| 57 | PAICS |  |  | 2407 | 2.255 | 0.4669 | Yes |
| 58 | WSB2 |  |  | 2459 | 2.219 | 0.4699 | Yes |
| 59 | EBNA1BP2 |  |  | 2478 | 2.211 | 0.4754 | Yes |
| 60 | TXNL2 |  |  | 2484 | 2.208 | 0.4819 | Yes |
| 61 | HMGB1 |  |  | 2520 | 2.191 | 0.4861 | Yes |
| 62 | CDK2 |  |  | 2688 | 2.099 | 0.4799 | Yes |
| 63 | PSMD2 |  |  | 2759 | 2.063 | 0.4810 | Yes |
| 64 | HSPC111 |  |  | 2760 | 2.062 | 0.4874 | Yes |
| 65 | PFN1 |  |  | 2943 | 1.961 | 0.4797 | No |
| 66 | UMPS |  |  | 3124 | 1.877 | 0.4718 | No |
| 67 | RNASEH2A |  |  | 3126 | 1.876 | 0.4776 | No |
| 68 | IL7R |  |  | 3145 | 1.868 | 0.4820 | No |
| 69 | MTHFD1 |  |  | 3258 | 1.814 | 0.4792 | No |
| 70 | EEF1E1 |  |  | 3449 | 1.730 | 0.4701 | No |
| 71 | PDAP1 |  |  | 3533 | 1.693 | 0.4691 | No |
| 72 | CKLF |  |  | 3625 | 1.651 | 0.4673 | No |
| 73 | RBMX |  |  | 3634 | 1.647 | 0.4718 | No |
| 74 | BRIP1 |  |  | 3774 | 1.596 | 0.4662 | No |
| 75 | C11ORF24 |  |  | 3831 | 1.578 | 0.4668 | No |
| 76 | PA2G4 |  |  | 3866 | 1.566 | 0.4691 | No |
| 77 | FLNC |  |  | 3906 | 1.550 | 0.4710 | No |
| 78 | UAP1 |  |  | 3959 | 1.529 | 0.4718 | No |
| 79 | POLE2 |  |  | 3975 | 1.520 | 0.4754 | No |
| 80 | TPM1 |  |  | 4482 | 1.324 | 0.4411 | No |
| 81 | TNFRSF12A |  |  | 4486 | 1.323 | 0.4449 | No |
| 82 | MAP3K8 |  |  | 4547 | 1.304 | 0.4444 | No |
| 83 | MRPL12 |  |  | 4601 | 1.286 | 0.4444 | No |
| 84 | LOXL2 |  |  | 4661 | 1.265 | 0.4439 | No |
| 85 | PLOD2 |  |  | 4888 | 1.192 | 0.4304 | No |
| 86 | PSMA7 |  |  | 4890 | 1.191 | 0.4340 | No |
| 87 | HNRPR |  |  | 4913 | 1.185 | 0.4360 | No |
| 88 | DLEU1 |  |  | 4922 | 1.183 | 0.4391 | No |
| 89 | HAS2 |  |  | 5204 | 1.079 | 0.4211 | No |
| 90 | F3 |  |  | 5288 | 1.053 | 0.4181 | No |
| 91 | TCEB1 |  |  | 5401 | 1.010 | 0.4127 | No |
| 92 | MAPRE1 |  |  | 5601 | 0.944 | 0.4006 | No |
| 93 | PSMD12 |  |  | 5876 | 0.852 | 0.3824 | No |
| 94 | PCSK7 |  |  | 5893 | 0.849 | 0.3838 | No |
| 95 | GPLD1 |  |  | 6249 | 0.730 | 0.3592 | No |
| 96 | SSR3 |  |  | 6643 | 0.609 | 0.3312 | No |
| 97 | SFRS2 |  |  | 6677 | 0.597 | 0.3306 | No |
| 98 | IMP4 |  |  | 6728 | 0.579 | 0.3286 | No |
| 99 | PSMC3 |  |  | 6733 | 0.577 | 0.3301 | No |
| 100 | BCCIP |  |  | 6816 | 0.546 | 0.3255 | No |
| 101 | DUT |  |  | 6839 | 0.539 | 0.3256 | No |
| 102 | IFRD2 |  |  | 6869 | 0.528 | 0.3250 | No |
| 103 | SMS |  |  | 6922 | 0.512 | 0.3226 | No |
| 104 | C13ORF1 |  |  | 6963 | 0.501 | 0.3212 | No |
| 105 | LSM4 |  |  | 7047 | 0.470 | 0.3163 | No |
| 106 | PITPNC1 |  |  | 7283 | 0.390 | 0.2997 | No |
| 107 | LSM3 |  |  | 7611 | 0.266 | 0.2757 | No |
| 108 | PNN |  |  | 7636 | 0.256 | 0.2747 | No |
| 109 | DLEU2 |  |  | 7765 | 0.204 | 0.2656 | No |
| 110 | NME1 |  |  | 7807 | 0.187 | 0.2631 | No |
| 111 | NUP107 |  |  | 8113 | 0.067 | 0.2401 | No |
| 112 | MT3 |  |  | 8224 | 0.025 | 0.2319 | No |
| 113 | PLG |  |  | 8307 | -0.001 | 0.2256 | No |
| 114 | TAGLN |  |  | 8576 | -0.113 | 0.2057 | No |
| 115 | VIL2 |  |  | 8844 | -0.228 | 0.1861 | No |
| 116 | NUPL1 |  |  | 8850 | -0.231 | 0.1864 | No |
| 117 | DCK |  |  | 8935 | -0.280 | 0.1809 | No |
| 118 | ID3 |  |  | 8993 | -0.308 | 0.1776 | No |
| 119 | ID2 |  |  | 9049 | -0.335 | 0.1744 | No |
| 120 | MKKS |  |  | 9066 | -0.345 | 0.1743 | No |
| 121 | JTV1 |  |  | 9145 | -0.379 | 0.1695 | No |
| 122 | NOLA2 |  |  | 9385 | -0.499 | 0.1530 | No |
| 123 | SDC1 |  |  | 9416 | -0.521 | 0.1523 | No |
| 124 | CBX1 |  |  | 9417 | -0.522 | 0.1539 | No |
| 125 | HNRPA2B1 |  |  | 9531 | -0.578 | 0.1471 | No |
| 126 | TUBG1 |  |  | 9551 | -0.592 | 0.1475 | No |
| 127 | SNRPE |  |  | 10109 | -0.910 | 0.1081 | No |
| 128 | TFPI2 |  |  | 10754 | -1.356 | 0.0634 | No |
| 129 | HMGN2 |  |  | 10916 | -1.492 | 0.0558 | No |
| 130 | RBM14 |  |  | 10941 | -1.521 | 0.0587 | No |
| 131 | MRPS28 |  |  | 10958 | -1.537 | 0.0623 | No |
| 132 | COPS6 |  |  | 11138 | -1.691 | 0.0540 | No |
| 133 | GNG11 |  |  | 11366 | -1.898 | 0.0426 | No |
| 134 | MYCBP |  |  | 11420 | -1.954 | 0.0447 | No |
| 135 | RNF41 |  |  | 11501 | -2.037 | 0.0449 | No |
| 136 | MRPS16 |  |  | 11541 | -2.078 | 0.0484 | No |
| 137 | SSSCA1 |  |  | 11818 | -2.347 | 0.0348 | No |
| 138 | MYL6 |  |  | 11843 | -2.362 | 0.0403 | No |
| 139 | IPO4 |  |  | 12777 | -3.812 | -0.0187 | No |
| 140 | MNAT1 |  |  | 12891 | -4.114 | -0.0145 | No |
| 141 | COX17 |  |  | 12981 | -4.414 | -0.0075 | No |
| 142 | POLR3K |  |  | 12995 | -4.441 | 0.0053 | No |
| 143 | EMP2 |  |  | 13273 | -6.228 | 0.0036 | No |
Table: GSEA details [plain text format]

  

Fig 2: CHANG\_SERUM\_RESPONSE\_UP: Random ES distribution      
 Gene set null distribution of ES for **CHANG\_SERUM\_RESPONSE\_UP**
